# Impact of desert dust storms, PM_10_ levels and daily temperature on mortality and emergency department visits due to stroke

**DOI:** 10.3389/fpubh.2023.1218942

**Published:** 2023-09-14

**Authors:** M. Murat Oktay, Behcet Al, Mustafa Boğan, Seval Kul, Hasan Gümüşboğa, Hasan Bayram

**Affiliations:** ^1^Emergency Department, School of Medicine, Gaziantep Islam Science and Technology University, Gaziantep, Türkiye; ^2^Department of Emergency Medicine, School of Medicine, Gaziantep University, Gaziantep, Türkiye; ^3^Emergency Department, School of Medicine, Istanbul Medeniyet University, Istanbul, Türkiye; ^4^Emergency Department, School of Medicine, Düzce University, Düzce, Türkiye; ^5^Biostatistics Department, School of Medicine, Gaziantep University, Gaziantep, Türkiye; ^6^Emergency Department, Şehitkamil State Hospital, Gaziantep, Türkiye; ^7^Department of Pulmonary Medicine, School of Medicine, Koç University, Istanbul, Türkiye; ^8^Department of Pulmonary Medicine, School of Medicine, Gaziantep University, Gaziantep, Türkiye

**Keywords:** desert dust storms, maximum temperature, stroke, emergency department visits, mortality

## Abstract

**Objective:**

It is known that the inhalation of air pollutants adversely affects human health. These air pollutants originated from natural sources such as desert storms or human activities including traffic, power generating, domestic heating, etc. This study aimed to investigate the impacts of desert dust storms, particulate matter ≤10 μm (PM_10_) and daily maximum temperature (MT) on mortality and emergency department (ED) visits due to stroke in the city of Gaziantep, Southeast Turkey.

**Method:**

The data on mortality and ED visits due to stroke were retrospectively recruited from January 1, 2009, to March 31, 2014, in Gaziantep City Centre.

**Results:**

PM_10_ levels did not affect ED visits or mortality due to stroke; however, MT increased both ED visits [adjusted odds ratio (OR) = 1.002, 95% confidence interval (CI) = 1.001–1.003] and mortality (OR = 1.006, 95% CI = 0.997–1.014) due to stroke in women. The presence of desert storms increased ED visits due to stroke in the total population (OR = 1.219, 95% CI = 1.199–1.240), and all subgroups. It was observed that desert dust storms did not have an increasing effect on mortality.

**Conclusion:**

Our findings suggest that MT and desert dust storms can induce morbidity and mortality due to stroke.

## Introduction

Stroke is a neurological condition that is seen because of the disruption in cerebral perfusion due to thromboembolic events, the majority of which are associated with ischemia. It causes significant mortality and morbidity. The most important cause of secondary injuries at the cellular level in stroke patients is the depletion of oxygen and glucose due to interrupted blood flow (1–3).

It is known that the inhalation of pollutants dispersed in the atmosphere adversely affects human health (4–10). High level of car traffic, increased combustion of fossil fuels for industrial activities, transport, and domestic heating lead to elevated levels of air pollutants including particulate matter (PM) levels in the atmosphere (4). Air pollution causes human health problems such as respiratory, cardiovascular, cerebrovascular, and obstetric diseases (4, 5, 7–11). Basic biological mechanisms that explain the effects of air pollutants on vascular structures are still controversial (3). The severity of the effects of this condition on human health varies depending on the type of pollutant, its concentration in the atmosphere, and the duration of its stay in the atmosphere.

Climate change is a global health concern, and the Intergovernmental Panel on Climate Change (IPCC) reported globally averaged greenhouse gas concentrations, along with land and ocean surface temperatures, have strikingly increased across the past decades (12). These increased temperatures have led to global warming resulting in increased risk for extreme meteorological and environmental events including extreme temperatures and desert dust storms (8, 12, 13). Epidemiological studies suggest an association between increased temperatures, and desert dust storms and morbidity and mortality (6, 10, 12, 14). People with pre-existing chronic diseases are at greater risk for increased adverse health outcomes (8), with vulnerable populations and people in low-middle income countries at a high level of susceptibility (10, 15).

Gaziantep is located in Southeast Turkey, close to the Syrian border. It is one of the most heavily polluted cities in Turkey (16), with the city under increased risk of desert dust storms, as is also experienced in other Southeastern Anatolian provinces in Turkey (17). The annual mean concentration of inhalable PM with a diameter ≤ 10 μm (PM_10_) in all Turkish city centres in 2000 was 49 μg/m^3^, and even higher in Gaziantep (55 μg/m^3^) (16). The PM with a diameter ≤ 2.5 μm (PM_2.5_) annual exposure for Gaziantep is reported as 66 μg/m^3^, which is 6.6 × 10 μg/m^3^ WHO guideline (18). The city of Gaziantep is at risk of desert dust storms like the other South-eastern Anatolian provinces in Turkey (17). Gaziantep and these provinces in the Southeast Turkey are under the effects of dust storms originating from deserts located in the Middle East’s Syrian Desert and Africa’s Saharan Desert (13, 17, 19). Studies reported that the frequency of desert dust storms has increased in the Middle East (20).

Recently, we have investigated effects of desert dust storms, PM_10_ and maximum temperature on morbidity and mortality due to cardiovascular and pulmonary diseases, and we found that desert dust storms, PM_10_ levels and maximum temperature were associated with increased mortality and ER visits due to acute coronary syndrome, asthma, COPD, lower respiratory tract infections and pulmonary emboli (5, 8). The current study aimed to investigate the impact of desert dust storms, PM_10_ levels and maximum temperature on mortality and emergency department (ED) visits due to stroke in the city of Gaziantep, Southeast Turkey.

## Methods

The study was conducted retrospectively in Gaziantep after obtaining the approval of the ethics committee (23.06.2014/228). The study was carried out in accordance with the Declaration of Helsinki.

### Health data

Health data were collected from three hospitals in Gaziantep city. The city-affiliated mortality rates were collected from the Ministry of Health. Data collected in 1916 days, from January 1, 2009, to March 31, 2014, were evaluated in this study. Collected data were analysed within the total population and in four separate subgroups. Subgroups were based on gender and age groups of individuals above and below 65 (≥65 & <65) years of age. Patients over 16 years of age admitted to the ED with a diagnosis and cause of death associated directly with a stroke were included in the study. Patients who could be diagnosed with stroke by clinical examination and radiological imaging modalities (magnetic resonance imaging, MRI or computed tomography, CT) were included in the study. Haemorrhagic strokes were excluded.

### Climate and particulate matter pollution data

The highest, the lowest, and average temperature values, as well as humidity, air pressure, and wind velocity data for the relevant dates (01, 2009 to March 31, 2014), were obtained from the General Directorate of Meteorology. Information daily on PM_10_ values in Gaziantep was obtained from the Air Quality Laboratory of the Ministry of Environment and Urbanization. Information regarding daily desert dust storms within the dates of the study period was obtained from the website owned and operated by the NASA.^1^ Daily aerosol optical depth (AOD) over land and ocean mean values were obtained for the coordinates of Gaziantep City. AOD values indicate how much direct sunlight is prevented from reaching the ground by these aerosol particles. An increased AOD value indicates more dust and haze in the atmosphere (21, 22). As reported by the National Oceanic and Atmospheric Administration, an AOD of 0.01 corresponds to an extremely clean atmosphere, and a value of 0.4 corresponds to a very hazy condition. In the present study, we used an AOD value >0.5 as an indicator of a desert dust storm day (5, 7, 8). Additionally, the presence of desert dust storm days was also confirmed by the records of Gaziantep Airport, Gaziantep, Turkey.

### Statistical analysis

Normal distribution of the data was tested using the Shapiro–Wilk test. The Student’s *T*-Test and Mann–Whitney U Test were used in the comparison of two independent groups of variables with normal or without normal distribution, respectively. Generalized Adaptive Additive Poisson Regression models (23, 24) were utilized to analyse the impact of the daily effect of PM_10_ levels, and the lag effect in the subsequent 3 days (lag0 = day zero; lag1 = day one; lag2 = day two, lag3 = day three of high PM_10_ levels, respectively), daily maximum temperature (MT0 = day zero (0) of maximum temperature levels), and presence of dust storm (DS0 = day zero (0) of dust storms) on ED visits and mortality due to stroke. Log link family was used for the smoothing function and penalized smoothing splines were used to adjust for seasonal patterns and long-term trends in disease morbidity adding time as a smoothing variable. All univariate statistical analyses were performed by SPSS for Windows (version 24.0) and generalized additive Poisson regression models were applied by mgcv package in R (version 3.4.1) for generalized additive modelling (GAM). The GAM command in mgcv was applied to solve the smoothing parameter estimation problem by using the generalized cross-validation (GCV) criterion. The best degree of freedom was automatically selected by GCV based on the Un-Biased Risk Estimator (UBRE) criterion. Adjusted odds ratios (ORs) and 95% confidence interval (CI) estimates were calculated to show the direction of the effects.

## Results

**Figure 1:**
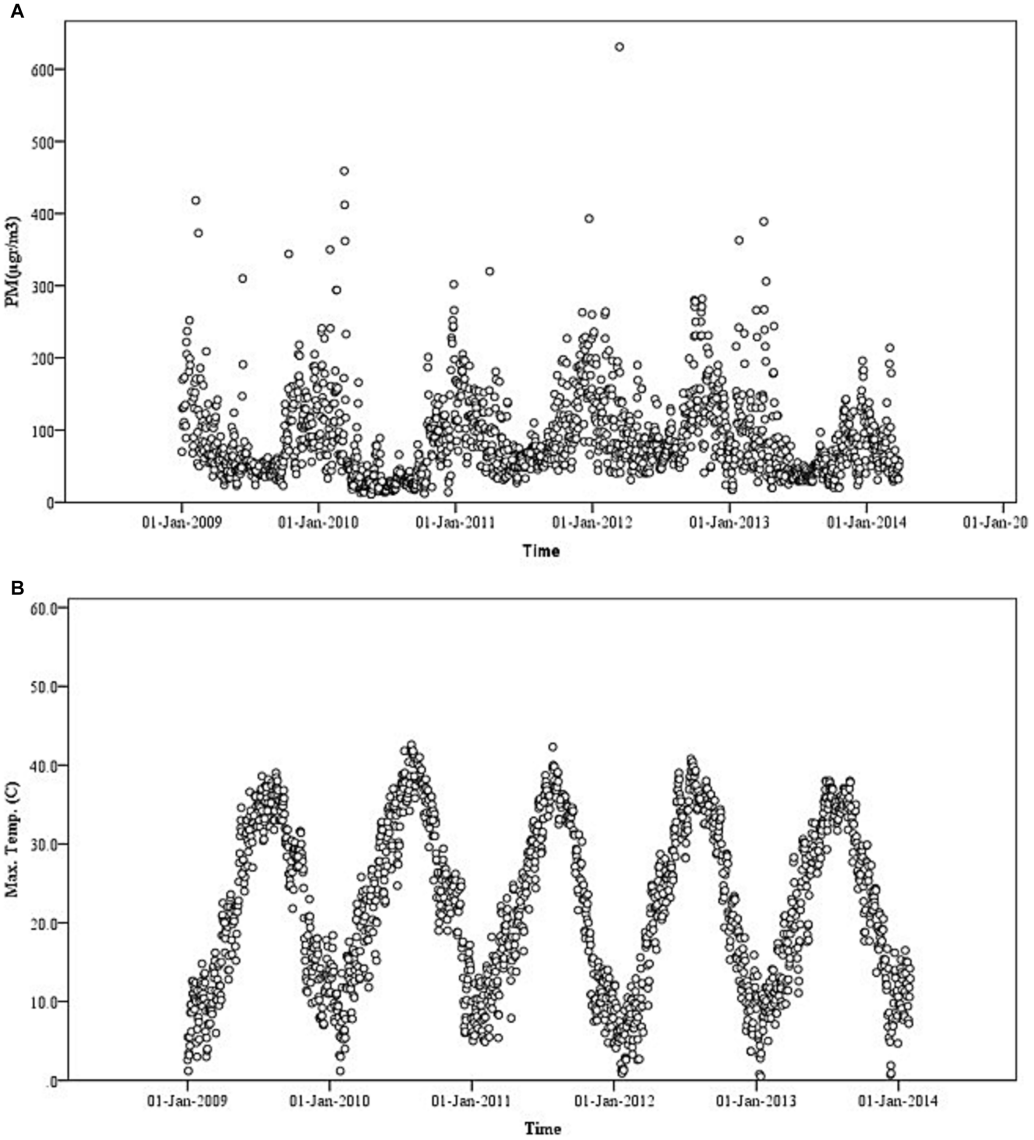
Daily mean of **(A)** particulate matter ≤10 μm (PM_10_) levels and **(B)** mean temperature (°C) in Gaziantep City Centre from September 01, 2009 to January 31, 2014 (5).

It was found that 89 dust storms occurred during the time of the study. Mean, minimum, and maximum temperature values were significantly higher (*p* = 0.001) on dusty days in comparison to dust-free days, and this comparison is provided in Table 1. Contrarily, mean air pressure (mbar) values were higher on dust-free days as compared to dusty days (*p* = 0.001). There was no significant difference between PM_10_ levels and relative humidity in dusty days, as compared to dust free days. PM_10_ levels (Figure 1A) and maximum temperatures (Figure 1B) for Gaziantep are provided in Figure 1.

**Table 1:** Descriptive statistics for pollutants and meteorological variables.

Variables median (IQR)	Overall (*n* = 1,916 days)	Dust storms present (*n* = 89 days)	No dust storms (*n* = 1,827 days)	*p*
PM (μg/m^3^)	72 [48–115]	74 [53.25–135]	72 [48–114]	0.227
Mean temperature (°C)	14.5 [7.6–24.5]	21.8 [16.7–27]	13.9 [7.3–24.2]	0.001*
Maximum temperature (°C)	21.8 [12.6–31.8]	28.55 [23.42–34.57]	21.2 [12.4–31.6]	0.001*
Minimum temperature (°C)	9.1 [4–17.8]	16.1 [10.8–21.3]	8.7 [3.9–17.6]	0.001*
Relative humidity	60.3 [41–79]	53 [40.7–77.3]	60.85 [41–79.3]	0.288
Air pressure (mbar)	915.7 [912.3–919.75]	913.4 [910.8–916.05]	915.9 [912.4–919.9]	0.001*

IQR, Inter-quartile range; *Significant at 0.05 level; Mann Whitney U test.

Approximately 10,000,000 ED visits were reviewed, and stroke was detected in 65,429 patients (6.54%). Of these patients, females and males were 36,056 (55.10%) and 29,373 (44.9%), respectively. The mean age of the ED visited patients was 61.36 years (females = 61.33 years and males = 61.39 years). Of the patients included in the study, 33,205 (51.79%) were below 65 years. Of stroke patients, 64,113 (98%) were discharged, and 1,316 (2%) patients died after ED visit (females = 729, 55.39% and males = 587, 44.61%). The mean age of the patients, who died was 73.45 years (females = 75.24 years and males = 71.66 years). Among patients died, 257 (19.52%) were below 65 years.

**Figure 2:**
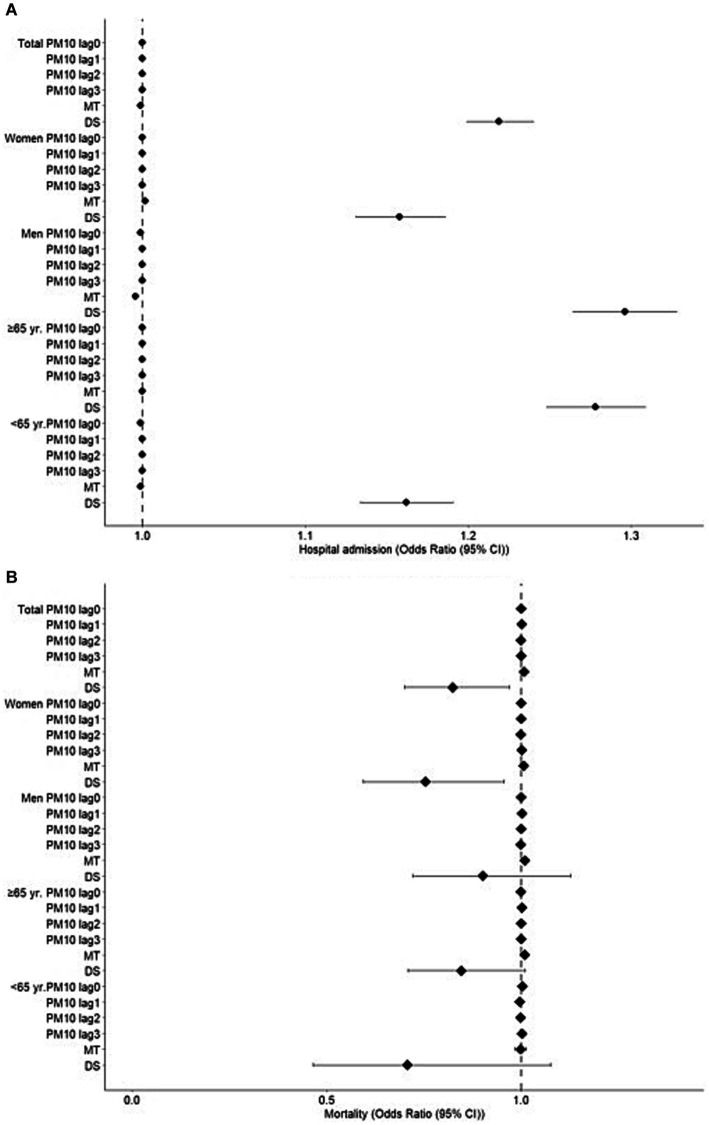
Effects of particulate matter ≤10 μM (PM_10_, at a lag time of 0–3 days), maximum temperatures (MT) and desert dust storms (DS) on stroke. Results of generalized additive Poisson models for predicting number of emergency department (ED) visits and deaths due to stroke.

PM_10_ levels and residual effects (lag0-3) did not have an effect on the mortality due to stroke. However, we observed that maximum temperature increased mortality (OR = 1.007, 95% CI = 1.001–1.013), while leading to a slight decrease in the number of ED visits in the total population (OR = 0.999, 95% CI = 0.998–1.000). Maximum temperature was associated with increased mortality due to stroke in total population (OR = 1.007, 95% CI = 1.001–1.013). Similarly, there was an association between maximum temperature and both mortality (OR = 1.006, 95% CI = 0.997–1.014) and ED visits (OR = 1.002, 95% CI = 1.001–1.003) due to stroke in women. Although desert dust storms did not increase mortality, they were associated with an increase in ED visits due to stroke in the total population (OR = 1.219, 95% CI = 1.199–1.240) and subgroups of women (OR = 1.158, 95% CI = 1.131–1.186) and men (OR = 1.296, 95% CI = 1.264–1.328). The results presenting the association between PM_10_, maximum temperature and desert dust storms and ED visits and mortality due to stroke are shown in Table 2 and Figure 2.

**Table 2:** Results of generalized additive Poisson models for predicting number of emergency department visits and mortality due to stroke.

Variables		ED visits OR [95% CI]^a^		Mortality OR [95% CI]^a^	
Total (*n* = 211.352)	PM_10_ lag0	1.000	[0.999–1.000]	1.000	[0.998–1.001]
	PM_10_ lag1	1.000	[1.000–1.000]	1.001	[0.999–1.003]
	PM_10_ lag2	1.000	[1.000–1.000]	0.999	[0.998–1.001]
	PM_10_ lag3	1.000	[1.000–1.000]	1.000	[0.999–1.002]
	MT	0.999	[0.998–1.000]	1.007	[1.001–1.013]
	DS	1.219	[1.199–1.240]	0.824	[0.700–0.969]
Women (*n*=138.536)	PM_10_ lag0	1.000	[0.999–1.000]	1.000	[0.998–1.002]
	PM_10_ lag1	1.000	[1.000–1.001]	1.000	[0.997–1.002]
	PM_10_ lag2	1.000	[0.999–1.000]	0.999	[0.997–1.002]
	PM_10_ lag3	1.000	[1.000–1.000]	1.001	[0.999–1.003]
	MT	1.002	[1.001–1.003]	1.006	[0.997–1.014]
	DS	1.158	[1.131–1.186]	0.754	[0.594–0.956]
Men (*n* = 72.816)	PM_10_ lag0	0.999	[0.999–1.000]	1.000	[0.998–1.002]
	PM_10_ lag1	1.000	[1.000–1.000]	1.002	[1.000–1.004]
	PM_10_ lag2	1.000	[0.999–1.000]	1.000	[0.997–1.002]
	PM_10_ lag3	1.000	[0.999–1.000]	0.999	[0.997–1.002]
	MT	0.996	[0.994–0.997]	1.009	[0.999–1,018]
	DS	1.296	[1.264–1.328]	0.901	[0.721–1.126]
≥ 65 yr.	PM_10_ lag0	1.000	[0.999–1.000]	0.999	[0.997–1.001]
	PM_10_ lag1	1.000	[1.000–1.000]	1.002	[1.000–1.004]
	PM_10_ lag2	1.000	[1.000–1.000]	1.000	[0.998–1.002]
	PM_10_ lag3	1.000	[0.999–1.000]	1.000	[0.998–1.002]
	MT	1.000	[0.998–1.001]	1.009	[1.002–1.016]
	DS	1.278	[1.248–1.309]	0846	[0.709–1.010]
<65 yr.	PM_10_ lag0	0.999	[0.999–1.000]	1.003	[1.000–1.006]
	PM_10_ lag1	1.000	[1.000–1.001]	0.996	[0.992–1.001]
	PM_10_ lag2	1.000	[0.999–1.000]	0.998	[0.993–1.003]
	PM_10_ lag3	1.000	[1.000–1.000]	1.002	[0.998–1.005]
	MT	0.999	[0.998–1.000]	0.998	[0.984–1.013]
	DS	1.162	[1.134–1.191]	0.707	[0.465–1.075]

ED, Emergency department; DS, Dust storm.

a Adjusted odd ratios and 95% confidence intervals.

## Discussion

In the present study, we investigated the effects of PM_10_ pollution, desert dust storms, and daily temperatures on mortality and morbidity due to stroke, in Southeast Turkey. Although PM_10_ levels did not associate with ED visits or mortality due to stroke, desert dust storms were positively associated with ED visits. Similarly, maximum temperature had a positive association with stroke mortality in the study population, and ED visits in women. Furthermore, maximum temperature was associated with increased mortality in only patients ≥65 years old. These findings suggest that desert dust storms and higher daily temperatures can increase the risk for mortality and ED visits due to stroke.

Nearly 3 million people die each year due to ischemic heart disease or ischemic stroke associated with air pollution rather than conventional risk factors such as obesity, diabetes, and smoking, wherein air pollution also increases the symptoms of sensitive individuals who have a chronic disease (5, 25). The relationship between desert dust storms, which is a natural source of air pollution, and stroke-related ED visits and stroke-related mortality has been demonstrated in various studies (26–28). The fact that dust storms, which were shown to increase the prevalence of ischemic stroke, not only contain PM originating from soil and other earth sources but also contain microorganisms such as bacteria and fungi, acidic gases, plant pollen, and various minerals suggesting that such health effects should not only be associated with the core PM levels (29–32).

Studies investigating the impact of desert dust storms on cerebral mortality and morbidity have reported positive associations. In a study investigating the association between dust storms and county-level non-accidental mortality in the United States from 1993 to 2005, Crook et al., found that total non-accidental mortality increased by 7.4% in the whole country (26). Mallone et al., reported a significant relationship between dust storms and cerebrovascular mortality in Italy (27). In their study on Asian desert dust storms, Yorifuji and Kashima investigated the Asian dust and suspended particulate matter and mortality due to stroke, and they found a positive relationship between dust storms and ischemic and haemorrhagic stroke in 47 cities in Japan (28). According to this study, a 10 μg/m^3^ increase of dust particles were positively associated with mortality due to intracerebral haemorrhage and ischemic stroke. However, our findings did not show a positive association between dust storms and stroke mortality. In contrast, there was an inverse relation between dust storms and mortality. Although we do not have a clear explanation for this discrepancy, we still think that this may be related to the recording of the last cause of death in the data obtained from the cemetery’s institution.

Studies also suggest an association between desert dust and morbidity due to stroke. In their research studying the association between Asian dust storms and ischemic stroke, Kamouchi et al., did not find a significant association with the incidence of ischemic stroke; however, Asian dust was significantly associated with the incidence of atherothrombotic brain infarction (33). Similarly, Yang et al., reported an increase in the number of visits due to stroke during dust storm days, whereas there was no significant relationship in the subsequent 3 days following the storm (34). However, they found that the risk of haemorrhagic stroke was higher than that of ischemic stroke (34). In a study of more than 800,000 were hospitalized because of stroke, Kang et al., found an increase in the number of stroke cases on the 1st and 2nd days following the Asian dust storms (35). In a recent study from Iran, dust storms were related with an increase in the risk of stroke (36). In agreement with these studies, we demonstrated an association between desert dust storms and increases in the number of ED visits due to ischemic stroke.

The majority of studies on PM levels and cerebral diseases suggested a positive relations (37–45); however, some failed to show such a relationship (46). In a study conducted on 7,594 patients, it was found that a 10 μg/m^3^ increase in PM_10_ levels was associated with increased cerebrovascular mortality (42). Similarly, Yan et al., showed that short-term exposure to PM_10_ led to an increase in stroke-related mortality, which peaked at lag1 (47). In a meta-analysis by Scheers et al., the authors found a significant association between exposure to PM_10_ and PM_2.5_ and increased hazard ratio for overall stroke events and stroke mortality, respectively (2).

Similarly, studies of particulate pollution reported positive associations with increases in cerebrovascular morbidity. In a study by Xiang et al., it was shown that each 10 μg/mm^3^ increase in PM_10_ levels resulted in a 1% increase in the number of daily admissions due to stroke (39). Alimohammadi et al., also found a correlation between the increases in PM levels and the number of patients presented to emergency room because of stroke (40). The prevalence of ischemic stroke was observed to increase on lag0-2 days following PM_10_ elevation (41). In a study of 63,997 stroke cases, PM_10_ exposure was shown to have a nonlinear but delayed relationship with stroke (43). Women and individuals over the age of 65 years were more affected by PM_10_ exposure (43). Chen et al., showed that PM_10_ exposure was cumulatively effective in increasing stroke-related hospitalizations by 4.1% at lag0 and lag1 days (44). Zhang et al., showed that every 10 μg/m^3^ increments in PM_10_ level increased stroke-related ED admissions (45). In that study, 65,429 patients were admitted and of those, 1,316 patients died. More recently, in single-pollutant model, each 10 μg/m^3^ increase in exposure to PM_10_ was associated with increase in OR for hospital admissions due to recurrent ischemic cerebrovascular events (48). Similarly, levels of PM_10_ were significantly associated with acute cerebrovascular events in a study collecting data of 2,534 days in a large metropolitan area (49). In contrast to these reports, we could not detect an association between PM_10_ levels and mortality or ED visits due to stroke. In subgroup analysis, there was no association in women or older adult, either.

Evidence suggests an impact of changes in ambient temperature on cerebrovascular diseases, and both low (50) and higher (51) temperatures reported to induce cerebrovascular mortality. It was also found that although PM_10_ had an increasing effect on stroke events in cold seasons, this became non-significant in warm seasons suggesting that temperatures may modify adverse health effects of air pollutants (39). Interestingly, increased average daily temperatures reduced the risk of stroke in male population in a recent study from Iran. The authors speculated that high average temperatures could be protective on the risk of Sadeghimoghaddam et al. (36). Although it was shown that meteorological changes (temperature, humidity, and pressure) could influence haemorrhagic stroke, these changes did not have any effect on ischemic stroke (6). In our study population, increased maximum temperatures were associated with mortality due to stroke in the general population, as well as in patients above 65 years. And although we could not find a relation between maximum temperature and visit due to stroke in the general population, we observed an association between maximum temperature and increased ED stroke visits in females, while this was decreased in males. We think that the increase in air temperature as well as clinical conditions such as tachycardia, increased systemic arterial pressure, hyperthermia, and fluid and electrolyte imbalance displacement contributed to the mortality.

### Limitations of the study

The most significant limitation was the fact that this was a retrospective study.

## Conclusion

Our findings have demonstrated that PM_10_ pollution, desert dust storms and meteorological variables such as ambient temperature can adversely affect cerebrovascular mortality and morbidity in Southeast Turkey. Health authorities, national and local authorities should develop mitigation strategies and take preventive measures against global climate change and environmental problems such as air pollution.

## Data availability statement

The raw data supporting the conclusions of this article will be made available by the authors, without undue reservation.

## Ethics statement

Ethical review and approval was not required for the study on human participants in accordance with the local legislation and institutional requirements. Written informed consent from the participants was not required to participate in this study in accordance with the national legislation and the institutional requirement. Approval details from ethics committee: (23.06.2014/228).

## Author contributions

MO, BA, and HB participated in designing the study. MB, HG, and MO provided the meteorological and hospital data. SK performed statistical modeling and tabulation of results and wrote the first draft of the paper. MB, BA, and HB helped in writing the final manuscript and discussion of the results. All authors contributed to the article and approved the submitted version.

## Conflict of interest

The authors declare that the research was conducted in the absence of any commercial or financial relationships that could be construed as a potential conflict of interest.

## Publisher’s note

All claims expressed in this article are solely those of the authors and do not necessarily represent those of their affiliated organizations, or those of the publisher, the editors and the reviewers. Any product that may be evaluated in this article, or claim that may be made by its manufacturer, is not guaranteed or endorsed by the publisher.

## Footnotes

^1^ http://earthobservatory.nasa.gov/
